# Interpersonal violence against women and maternity care in Migori County, Kenya: evidence from a cross-sectional survey

**DOI:** 10.3389/fgwh.2024.1345153

**Published:** 2024-05-09

**Authors:** Sophie K. Schellhammer, Joseph R. Starnes, Sandra Mudhune, Lou Goore, Lauren Marlar, Samuel Oyugi, Jane Wamae, Constance S. Shumba, Ash Rogers, Julius Mbeya, Beffy Vill, Angeline S. Otieno, Richard G. Wamai, Lawrence P. O. Were

**Affiliations:** ^1^ Vanderbilt University School of Medicine, Nashville, TN, United States; ^2^ Department of Pediatrics, Division of Pediatric Cardiology, Vanderbilt University School of Medicine, Nashville, TN, United States; ^3^ Lwala Community Alliance, Rongo, Kenya; ^4^ Division of Epidemiology and Social Sciences, Institute for Health and Equity, Medical College of Wisconsin, Milwaukee, WI, United States; ^5^ Department of Health Services, Migori County, Kenya; ^6^ Department of Cultures, Societies, and Global Studies, Northeastern University, Boston, MA, United States; ^7^ Department of Health Sciences & Department of Global Health, Boston University, Boston, MA, United States

**Keywords:** interpersonal violence, gender-based violence, maternal health, perinatal care, Kenya

## Abstract

**Background:**

Interpersonal violence (IPV) is an issue of major public health concern, with 24% of Kenyan women reporting physical violence perpetrated by a current husband or partner. IPV has profound impacts on physical and mental health outcomes, particularly for pregnant women; it has been found to increase the risk of perinatal mortality, low birth weight, and preterm birth. This study aims to identify variables associated with IPV and assess the effects of IPV experience on prenatal and peripartum maternal healthcare in Migori County, Kenya. Findings build on a previous study that investigated a smaller region of Migori County.

**Methods:**

Responses to cross-sectional household surveys conducted in six wards of Migori County, Kenya in 2021 from female respondents aged 18 and older were analyzed. The survey contained validated screening tools for interpersonal violence. Group-wise comparisons, and bivariate and multivariate logistic regression analyses were performed to describe community prevalence, factors associated with IPV against women, and the effect of IPV exposure on prenatal and peripartum health care.

**Results:**

This study finds that 2,306 (36.7%) of the 6,290 respondents had experienced lifetime IPV. IPV experience was associated with the age group 25–49 (adjusted odds ratio (aOR) 1.208; 95%CI: [1.045–1.397]; *p* = 0.011), monogamous marriage [aOR 2.152; 95%CI: (1.426–3.248); *p* < 0.001], polygamous marriage [aOR 2.924; 95%CI: (1.826–4.683); *p* < 0.001], being widowed/divorced/separated [aOR 1.745; 95%CI: (1.094–2.786); *p* < 0.001], feeling an attitude of “sometimes okay” toward wife beating [aOR 2.002 95%CI: (1.651, 2.428); *p* < 0.001], having been exposed to IPV in girlhood [aOR 2.525; 95%CI: (2.202–2.896); *p* < 0.001] and feeling safe in the current relationship [aOR 0.722; 95%CI: (0.609, 0.855); *p* < 0.001]. A depression score of mild [aOR 1.482; 95%CI: (1.269, 1.73); *p* < 0.001] and severe [aOR 2.403; 95%CI: (1.429, 4.039); *p* = 0.001] was also associated with IPV experience, and women who experienced emotional abuse were much more likely to have experienced IPV [aOR 10.462; 95% CI: (9.037, 12.112); *p* < 0.001]. Adjusted analyses showed that having experienced IPV was negatively associated with attending at least four antenatal care visits during the most recent pregnancy (OR 0.849, *p* = 0.044) and with having a skilled birth attendant (OR 0.638, *p* = 0.007).

**Conclusions:**

IPV is prevalent in Migori County, Kenya, with increased prevalence among women aged 25–49, those residing in West Kanyamkago, those in a monogamous or polygamous marriage, those who have been widowed/divorced/separated, and those with severe depressive symptoms. Further, IPV exposure is associated with lower use of maternal care services and may lead to worse maternal health outcomes. There is need for enhanced effort in addressing social and gender norms that perpetuate IPV, and this study can contribute to guiding policy interventions and community responses towards IPV.

## 1 Introduction

Interpersonal violence (IPV) is physical, sexual, or psychological harm perpetrated against another person (1). IPV perpetrated by an intimate partner and gender-based violence against women has been described by the World Health Organization (WHO) as a major public health problem that warrants the intervention of healthcare systems (2). Women are more likely to experience IPV; the United Nations’ Global Study on Homicide found that 82% of intimate partner homicide victims are female (3). The WHO estimates that 27% of women have experienced IPV in the form of physical or sexual abuse over the course of their lifetime (4).

To combat this issue, Goal 5 of the Sustainable Development Goals (SDGs) broadly aims to achieve gender equality. Target 5.2 specifically aims to eliminate violence against women by 2030 (5). The COVID-19 pandemic increased the urgency of this issue by increasing psychological and economic stressors globally. The isolation necessitated by the pandemic in many countries increased the vulnerability of women at risk for gender-based violence and made support services more difficult to access (6). Rates of interpersonal violence rose worldwide, including in China, India, and the United States (7–9).

IPV has profound impacts on health outcomes, beyond homicide alone. Women who have experienced IPV have increased emergency room, outpatient, inpatient, and mental health visits (10, 11). A history of IPV predisposes women to increased risk of disordered eating, physical trauma, sexually transmitted infections, HIV/AIDs, mental health issues such as depression, Post-Traumatic Stress Disorder (PTSD), suicidal ideation, and non-communicable diseases such as cardiovascular and gastrointestinal conditions (3, 12–16).

The negative health effects of IPV extend beyond the victim, especially in the case of pregnant women. IPV has been found to cause increased risk of perinatal mortality (17–20). It has also been associated with increased incidence of low birth weight and preterm birth (19, 21–24). Mothers experiencing IPV are less likely to attend their prenatal appointments and more likely to begin prenatal healthcare visits later into their pregnancy (19, 21–26). In addition to neonatal complications, children born to mothers who have experienced IPV may face long-term effects on their wellbeing, as maternal history of IPV has been found to impact the social-emotional development of their children (27). The experience of IPV may be cyclical for some children because growing up in a home where IPV is prevalent has been found to increase the risk of experiencing or perpetuating IPV in the future (28, 29).

In Kenya, the lifetime prevalence of IPV in women is estimated to be 38% by the WHO. This is 1.4 times higher than the global average (27%) and 1.15 times higher than the average in sub-Saharan Africa (33%) (4). Domestic violence is a leading cause of preventable deaths among young women in Kenya (30). According to Kenya's 2022 Demographic and Health Survey, 33.9% of women have experienced physical violence, and 13.0% of women have experienced sexual violence (31). A current husband or intimate partner perpetrated 53.9% of the physical violence and 70.9% of the sexual violence ever-married or partnered Kenyan women experienced (31).

Although Kenya has legal protections for these women, such as the Protection Against Domestic Violence Act of 2015, spousal rape continues not to be criminalized (32). IPV in Kenya has been associated with young marital age, low wealth index, urban residence, being 40–49 years of age, depression, minimal educational attainment, drug and alcohol abuse, and higher risk of contracting HIV infection (28, 33, 34). Kenyan women who have experienced IPV are less likely to attend antenatal care visits, less likely to deliver at a healthcare facility, and 40% less likely to access skilled delivery attendants during childbirth (35, 36). During COVID-19, rates of IPV in Kenya increased with sexual violence offences increasing by as much as 35% (32, 37).

According to Kenya's 2022 Demographic and Health Survey (DHS), out of 47 counties, Migori county in southwestern Kenya has the fourth highest proportion of women who reported physical violence (51.1%) and eighth highest proportion of women who reported sexual violence (16.7%) since the age of 15 (38). In the 2018 Kenya population-based HIV impact assessment, Migori county had the fourth highest HIV prevalence, at 13% (39). Although this question was not included on the 2022 DHS, the 2014 DHS showed that the women of Migori county have the lowest average age of first sexual intercourse (17.1 years) out of all Kenyan counties (38). The aforementioned variables have all been found to increase the risk of IPV (40, 41). To remedy IPV, community health workers (CHWs) in Kenya have been found to provide effective support, but there is room for improvement via training regarding IPV identification and prevention strategies (42, 43).

The Lwala Community Alliance (Lwala) is a non-governmental organization that serves to promote the health and well-being of communities in Migori County, Kenya. Lwala operates a health center in North Kamagambo and is working with the Migori County government to scale its community-led health model throughout the county. The model incorporates traditional birth attendants into professionalized community health worker cadres and is distinguished by its consistent payment, supportive supervision, and proactive community case finding and case management. To better understand community needs and measure the impact of programming, Lwala has conducted longitudinal cross-sectional community household surveys (44). The surveys include IPV assessment, which allowes for measurement of the prevalence of IPV in the Lwala catchment area. The goals of our study are to identify changes in IPV prevalence with the expansion of the survey's geographic reach, characterize variables associated with IPV, and to assess the effects of past IPV experience on maternal healthcare utilization. Such an analysis provides hyperlocal data to identify possible points of intervention to reduce IPV as well as provides justification for developing an array of timely interventions given IPV's negative impact on maternal outcomes in a setting with one of the highest HIV rates in the country.

## 2 Methods

### 2.1 Study setting

**Figure 1:**
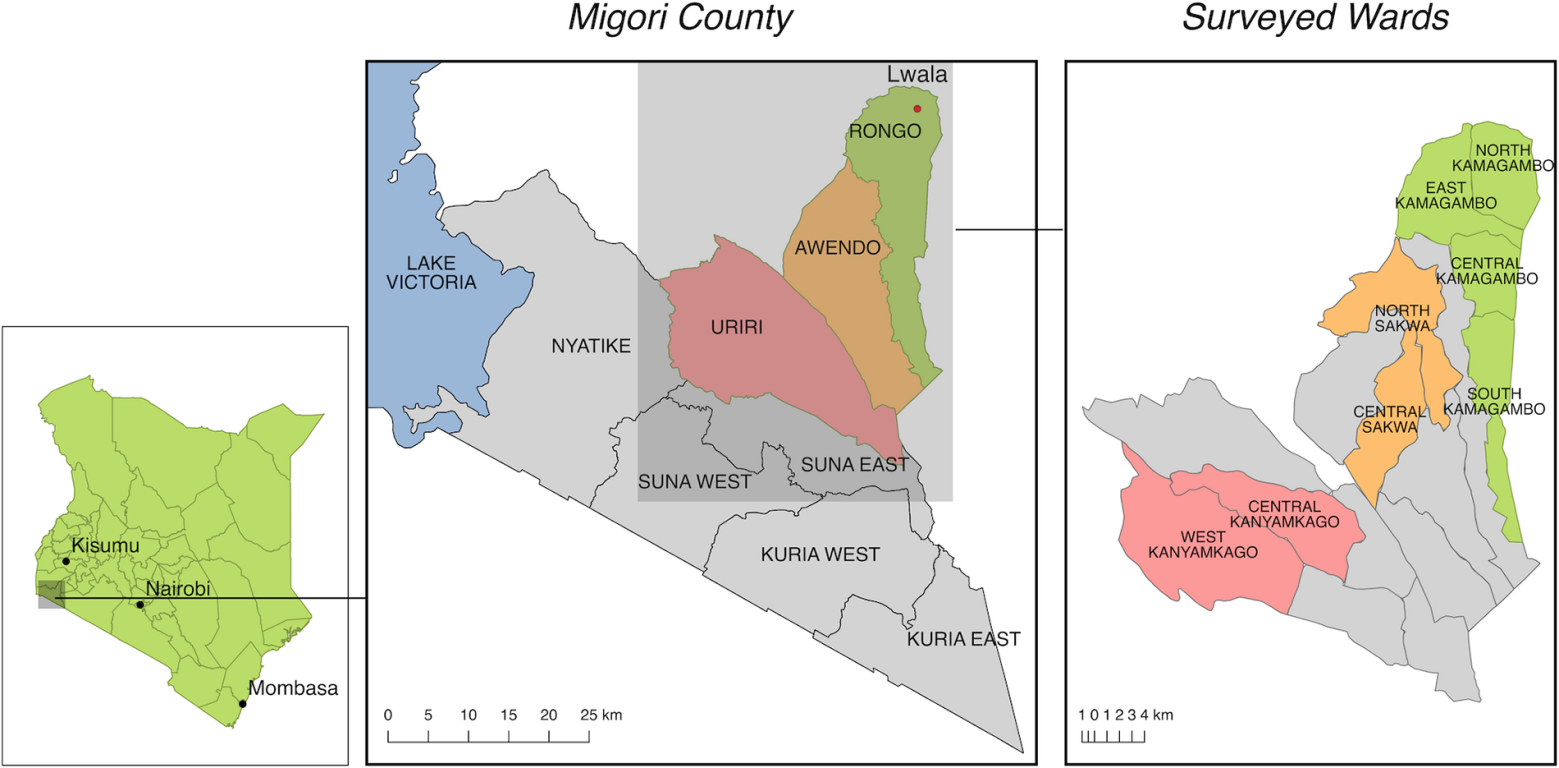
Migori county, Kenya. Lwala programming began in North Kamagambo in Rongo sub-county (green). At the time of the survey, all of Rongo except Central Kamagambo was receiving Lwala services. The next expansion is planned for Awendo (orange). Two areas in Uriri, Central Kanyamkago and West Kanyamkago, serve as comparison wards (red) (44).

Migori County (Figure 1) is located in western Kenya and has a population of approximately 1.1 million (45). The economy is primarily reliant on subsistence farming with fishing being prevalent in areas bordering Lake Victoria. In 2007, Lwala programming started in North Kamagambo in Rongo sub-county within Migori county. Since then, Lwala programming has expanded with additions in East Kamagambo in 2018, followed by South Kamagambo in 2019 both of which were surveyed in 2021. Also included in the 2021 survey was Central Kamagambo, where programming began after survey administration in 2021. Finally, the survey included two wards of Awendo sub-county intended for future programming (North Sakwa and Central Sakwa) and two nearby control areas without planned programming (Central Kanyamkago and West Kanyamkago).

### 2.2 Sampling and survey

The details of the sampling methodology and the resulting survey have been previously described in the survey protocol (44). To summarize, using a power of 80%, sample size was determined by the size required to identify a 10% change over time in each health metric. The survey was administered in 2017, 2019, 2021, and will be administered every three years until 2027 (44). A modified version of the World Health Organization Expanded Programme of Immunization (EPI) method was used for household selection (46, 47). Using Geographic Information Systems (GIS), each region was split into 127 grid squares. Enumerators arrived at the center of a square using Geographic Positioning Systems (GPS) and then spun a pen or bottle to randomly determine their starting direction. By using an arbitrary grid square's center, rather than a town center, as a starting point, possible bias of the traditional spin-the-bottle sampling method was reduced (48).

The cross-sectional, population-based survey used validated tools to reproducibly record numerous health metrics. To capture IPV metrics specifically, survey questions were adapted from two clinically validated screening tools for partner violence: the Abuse Assessment Screen (49), and the Partner Violence Screen (50), as well as from the Spousal Violence questionnaire found in the 2014 Kenya DHS. These captured and characterized experiences of violence in the community and in an intimate relationship, as well as attitudes toward such violence. IPV was defined as physical or sexual violence in the same manner as our previous work (28). Only data from female respondents were analyzed for partner violence. PHQ-8 was used to assess respondent mental health (51, 52). Depression severity, as measured by PHQ-8, was scored on a scale of none, mild, moderate, moderately severe, and severe. Additionally, demographics, highest level of education, health, and socioeconomic status of respondents and their households were captured by the survey. We assessed socioeconomic status by breaking participants into wealth quartiles using the multiple correspondence analysis methodology (53) similar to that used by the Kenya Demographic and Health Survey. The survey was administered using the Research Electronic Data Capture (REDCap) tool using electronic tablets (54, 55).

### 2.3 Statistical analysis

IPV was defined as being physically assaulted or forced to perform sexual acts by another person and defined based upon the aforementioned validated screening tools for interpersonal violence: Abuse Assessment Screen and the Partner Violence Screen (49, 50). This definition is consistent with Lwala Community Alliance's prior work on the topic. Descriptive statistics by IPV status were reported as counts and percentages for categorical variables and median with interquartile range (IQR) for continuous variables. A list of variables potentially associated with IPV was created *a priori* based on prior literature and organizational experience. Univariate logistic regressions were performed for each variable (Appendix Table A1), and a final multivariable logistic regression was performed to determine the association of these variables with the experience of IPV. Variables used in the multivariable logistic regression included region, age, religion, marital status, highest level of education, experience of emotional abuse, IPV exposure in girlhood, feeling safe in current relationship, history of HIV testing, depression severity, childhood mortality, attitudes supportive of wife beating, current pregnancy, and wealth quartile. Logistic regression, adjusted for the variables in the first analysis, was also performed to determine the association of IPV experience with perinatal health outcomes. All analyses were performed using Stata version 14.2 (StataCorp LP, College Station, TX).

### 2.4 Ethical approval

The protocol and study design were approved by the Ethics and Scientific Review Committee at AMREF Health Africa (AMREF-ESRC P452/2018) and the Institutional Review Board at Northeastern University (IRB #: 20-09-18). Informed consent was obtained from all participants prior to the survey. A research license was obtained from the Kenya National Commission for Science and Technology (NACOSTI/P/21/8776).

## 3 Results

### 3.1 Demographics

The total population included in this analysis was 6,290 females, 2,306 (36.7%) of whom had experienced IPV. The median age of all participating women was 27 years (Table 1). The majority (1,197, 78.6%) of women were in married, monogamous relationships. Religions practiced by participants included Seventh-day Adventist (SDA) (2,566, 40.8%), Catholic (979, 15.6%), Protestant (1,297, 20.1%), and Roho (1,063, 16.9%), while 622 (6.6%) of women responded with “other”. Only 90 (1.43%) women had no education, while the highest level of education attained by 3,371 (53.6%) women was primary school, and 2,829 (45.0%) had completed secondary school or higher.

**Table 1:** Demographics of respondents.

Variable	IPV-negative	IPV-positive	Total
Total	3,984 (63.3%)	2,306 (36.7%)	6,290
Region			
North Kamagambo	516 (64.5%)	284 (35.5%)	800
East Kamagambo	492 (61%)	315 (39%)	807
Central Kamagambo	525 (64.5%)	289 (35.5%)	814
South Kamagambo	506 (65%)	272 (35%)	778
Central Kanyamkago	488 (64.9%)	264 (35.1%)	752
West Kanyamkago	454 (58.1%)	327 (41.9%)	781
North Sakwa	520 (63.4%)	300 (36.6%)	820
Central Sakwa	483 (65.4%)	255 (34.6%)	738
Age (median, IQR)	27 (23, 32)	28 (24,34)	27 (23, 33)
Age category			
18–24	1,368 (69.3%)	607 (30.7%)	1,975
25–49	2,455 (60.6%)	1,595 (39.4%)	4,050
50+	161 (60.8%)	104 (39.3%)	265
Religion			
SDA	1,684 (65.6%)	882 (34.4%)	2,566
Catholic	609 (62.2%)	370 (37.8%)	979
Protestant	797 (62.9%)	470 (37.1%)	1,267
Roho	632 (59.5%)	431 (40.5%)	1,063
Other	262 (63.1%)	153 (36.9%)	415
Marital status			
Single	217 (82.5%)	46 (17.5%)	263
Married monogamous/cohabitating	3,145 (63.6%)	1,797 (36.4%)	4,942
Married polygamous	233 (50.3%)	230 (49.7%)	463
Widowed/divorced/separated	389 (62.5%)	233 (37.5%)	622
Highest level of education			
No education	49 (54.4%)	41 (45.6%)	90
Primary	2,026 (60.1%)	1,345 (39.9%)	3,371
Secondary+	1,909 (67.5%)	920 (32.5%)	2,829
Experience of emotional abuse			
No	3,612 (78.0%)	1,022 (22.1%)	4,634
Yes	372 (22.5%)	1,284 (77.5%)	1,656
IPV exposure in girlhood			
No	3,154 (73.2%)	1,157 (26.8%)	4,311
Yes	818 (41.8%)	1,138 (58.2%)	1,956
Feels safe in current relationship			
No	843 (66.8%)	419 (33.2%)	1,262
Yes	3,057 (62.6%)	1,823 (37.4%)	4,880
Ever HIV tested			
No	35 (70.0%)	15 (30.0%)	50
Yes	3,941 (63.3%)	2,289 (36.7%)	6,230
Depression score			
None	2,528 (69.9%)	1,087 (30.1%)	3,615
Mild	752 (53.1%)	665 (46.9%)	1,417
Moderate	369 (59.5%)	251 (40.5%)	620
Moderately severe	215 (56.0%)	169 (44.0%)	384
Severe	40 (36.0%)	71 (64.0%)	111
Childhood mortality (last 5 years)			
No	3,864 (63.3%)	2,240 (36.7%)	6,104
Yes	52 (61.9%)	32 (38.1%)	84
Attitude supportive of wife beating			
Never okay	3,637 (65.8%)	1,889 (34.2%)	5,526
Other (sometimes okay)	347 (45.4%)	417 (54.6%)	764
Currently pregnant			
No	3,587 (63.5%)	2,060 (36.5%)	5,647
Yes	387 (62.2%)	235 (37.8%)	622
Wealth quartile			
Severely poor	951 (60.5%)	622 (39.5%)	1,573
Poor	985 (62.7%)	587 (37.3%)	1,572
Vulnerable	960 (61.0%)	613 (39.0%)	1,573
Non-poor	1,088 (69.2%)	484 (30.8%)	1,572

This table shows the demographic breakdown of 6,290 women living in Migori County, Kenya who were surveyed in 2021 by the Lwala Community Alliance community household survey. The group of women was divided into those who had experienced IPV according to their survey responses (IPV-positive) and those who had not (IPV-negative).

Only 46 (17.5%) of the 263 single women surveyed had experienced IPV, which was the smallest group of IPV-positive women among all demographic characteristics analyzed. In four of the 46 analyzed variable groups, greater than 50% of respondents identified as IPV-positive: women who responded “yes” to experiencing emotional abuse (1,284, 77.5%), women who were exposed to IPV during girlhood (1,138, 58.2%), women who had a depression score of “severe” (71, 64.0%), and women who responded “other/sometimes okay” when asked about attitude toward wife beating (417, 54.6%).

### 3.2 Types of emotional and physical harm experienced by female survey respondents

Table 2 shows emotional and physical harm experienced by the women of Migori county at the hands of the general community, their family, and their partners. 1,874 (29.8%) women had been hit, kicked, punched, pushed, or otherwise hurt by someone in their family or in the community, while 434 (6.9%) women responded they had been involved in forced sexual activities.

**Table 2:** Types of emotional and physical harm experienced by respondents.

	N (%) yes
General questions (not partner specific)	
Hit, kicked, punched, pushed, or otherwise hurt by someone in your family or in the community^a^	1,874 (29.8%)
Forced sexual activities^a^	434 (6.9%)
Husband/partner specific questions	
Push you, shake you, or throw something at you?^a^	1,420 (22.6%)
Slap you or twist your arm?^a^	1,543 (24.5%)
Punch you with his fist or with something that could hurt you?^a^	943 (15.0%)
Kick you or drag you?^a^	643 (10.2%)
Try to strangle you or burn you?^a^	282 (4.5%)
Threaten you with a knife, gun or other type of weapon?	263 (4.2%)
Attack you with a knife, gun, or other type of weapon?^a^	231 (3.7%)
Physically force you to have sexual intercourse, even when you did not want to?^a^	318 (5.1%)
Force you to perform other types of sexual acts when you did not want to?^a^	276 (4.4%)
Say or do something to humiliate you in front of other people?	1,588 (25.3%)
Threaten you or someone close to you with harm?	1,004 (16.0%)
Insult you or make you feel bad about yourself?	1,478 (23.5%)
All are out of 6,290	

^a^ all marked items were included in this paper's definition of interpersonal violence.

This table shows the questions related to emotional abuse and IPV and the proportion of respondents who answered “yes” to these questions. The number of respondents who answered yes, as well as the percentage calculated by a simple fraction, are included.

As for husband and partner specific questions, the most reported harm experienced was a partner who humiliated their partner in front of others by saying or doing something (1,588, 25.3%). Other harmful scenarios that were experienced by greater than 20% of the 6,290 respondents included: being slapped or having an arm twisted (1,543, 24.5%), being insulted or made to feel bad about oneself (1,475, 23.5%), and being punched, shaken, or having an object thrown at them (1,420, 22.6%). The most seldom reported experience was an attack with knife, gun, or other weapon by the partner, which only 231 (3.7%) of respondents experienced. Items marked by an asterisk (*) in Table 2 are included as types of violence in our definition of IPV.

### 3.3 Factors associated with IPV

When compared to women aged 18–24 and adjusted for all other variables, women aged 25–49 were at increased odds of experiencing IPV, with an adjusted odds ratio (aOR) of 1.208 (95%CI 1.045–1.397, *p* = 0.011) (Table 3). Women who were in (1) married, monogamous relationships, (2) married, polygamous relationships, or (3) widowed, divorced, or separated had higher odds of experiencing IPV than single women (*p* < 0.001 for all). The odds of experiencing IPV in women who also experienced emotional abuse was 10.462 times higher (95%CI 9.037–12.112, *p* < 0.001) than in women who had not, and 2.525 times higher for women who had been exposed to IPV in girlhood [95%CI: (2.202–2.896); *p* < 0.001]. The odds of women who felt unsafe in their current relationship experiencing IPV was lower than those who felt safe in their relationship (aOR 0.722, 95%CI 0.609–0.855, *p* < 0.001). The odds of a respondent with a “severe” depression score experiencing IPV were higher than all other depression scores (aOR 2.403, 95%CI 1.429, 4.039, *p* = 0.001) when compared with women who had a depression score of “none”. In contrast to the reference group who deemed wife beating to be “never okay”, respondents who held a “sometimes okay” attitude toward wife beating were twice as likely to experience IPV (aOR 2.002, 95%CI 1.651–2.428, *p* < 0.001). Appendix Table A1 shows the crude odds ratios and *p*-values for the logistic regression.

**Table 3:** Multivariate regression of factors associated with IPV.

Variable	AOR (95% CI)	*P*-value
Region		
North Kamagambo	Ref	
East Kamagambo	1.246 (0.971, 1.598)	0.084
Central Kamagambo	0.855 (0.654, 1.119)	0.254
South Kamagambo	0.956 (0.739, 1.237)	0.732
Central Kanyamkago	0.928 (0.713, 1.207)	0.576
West Kanyamkago	1.093 (0.848, 1.41)	0.49
North Sakwa	1.051 (0.817, 1.351)	0.701
Central Sakwa	1.000 (0.77, 1.3)	0.998
Age		
18–24	Ref	
25–49	**1.208** (**1.045, 1.397)**	**0**.011*
50+	1.137 (0.766, 1.688)	0.524
Religion		
SDA	Ref	
Catholic	1.163 (0.96, 1.408)	0.124
Protestant	1.044 (0.874, 1.247)	0.633
Roho	1.209 (0.998, 1.465)	0.053
Other	1.076 (0.821, 1.41)	0.595
Marital status		
Single	Ref	
Married monogamous/cohabitating	**2.152** (**1.426, 3.248)**	**<0**.001***
Married polygamous	**2.924** (**1.826, 4.683)**	**<0**.001***
Widowed/divorced/separated	**1.745** (**1.094, 2.786)**	**0**.02*
Highest level of education		
No education	Ref	
Primary	1.199 (0.631, 2.28)	0.579
Secondary+	0.957 (0.497, 1.843)	0.896
Experience of emotional abuse		
No	Ref	
Yes	**10.462** (**9.037, 12.112)**	**<0**.001***
IPV exposure in girlhood		
No	Ref	
Yes	**2.525** (**2.202, 2.896)**	**<0**.001***
Feels safe in current relationship		
Yes	Ref	
No	**0.722** (**0.609, 0.855)**	**<0**.001***
Ever HIV tested		
No	Ref	
Yes	1.124 (0.557, 2.269)	0.744
Depression Score		
None	Ref	
Mild	**1.482** (**1.269, 1.73)**	**<0**.001***
Moderate	1.015 (0.812, 1.269)	0.896
Moderately severe	0.995 (0.759, 1.305)	0.972
Severe	**2.403** (**1.429, 4.039)**	**0**.001***
Childhood mortality (last 5 years)		
No	Ref	
Yes	1.29 (0.76, 2.189)	0.346
Attitude supportive of wife beating		
Never okay	Ref	
Sometimes okay	**2.002** (**1.651, 2.428)**	**<0**.001***
Currently pregnant		
No	Ref	
Yes	1.134 (0.918, 1.401)	0.242
Wealth quintile		
Non-poor	Ref	
Vulnerable	0.998 (0.826, 1.206)	0.982
Poor	0.842 (0.689, 1.028)	0.091
Severely poor	0.846 (0.682, 1.048)	0.125

This table includes the same variables as Table 1, but is a multivariate regression of those variables and their relationship with IPV. The adjusted odds ratio, as well as the 95% confidence interval are reported. *P*-values less than or equal to 0.05 are considered statistically significant.

Bold values indicate statistically significant findings.

* denotes *p* ≤ 0.05.

** denotes *p* ≤ 0.01.

*** denotes *p* ≤ 0.001.

### 3.4 Antenatal care visits

A total of 5,694 women (90.5%) had a child under five years of age and information available about antenatal care visits (ANC) (Table 4). Among these women, 4,390 (77.1%) attended at least four ANC visits during the pregnancy with this child. Adjusted for the variables in the IPV analyses, having experienced IPV was negatively associated with attending at least four antenatal care visits during the most recent pregnancy (OR 0.849, *p* = 0.044).

**Table 4:** Rates of antenatal care visits, skilled delivery, and facility delivery and multivariate regression of the association of prenatal and peripartum health care with IPV.

Antenatal care visits (ANC)		Skilled delivery		Facility delivery	
No. with complete data	5,694 (90.5%)	No. with complete data	5,641 (89.7%)	No. with complete data	5,667 (90.1%)
Attended at least 4 ANC visits	4,390 (77.1%)	Skilled birth attendant at delivery	5,397 (95.7%)	Delivered at a health facility	5,250 (92.6%)
AOR of IPV- positive women	0.849	AOR of IPV- positive women	0.638	AOR of IPV- positive women	0.846
*p*-value	**0.044** *	*p*-value	**0.007** **	*p*-value	0.196

This table describes the amount of women who had a child under five years of age and who had data regarding their number of ANC visits, whether there was a skilled birth attendant present at delivery, and whether they delivered their baby at a facility. These numbers all pertain to the women's most recent pregnancy. The number of women who met the metrics is listed, along with a simple fraction-derived percentage. Multiple logistic regression of the experience of IPV was performed for this group of women. *P*-values less than or equal to 0.05 were considered significant.

Bold values indicate statistically significant findings.

* denotes *p* ≤ 0.05.

** denotes *p* ≤ 0.01.

### 3.5 Skilled delivery

A total of 5,641 women (89.7%) had a child under five years of age and information available about skilled delivery attendance (Table 4). Among these women, 5,397 (95.7%) had a skilled birth attendant. Adjusted for variables in the IPV analyses, having experienced IPV was negatively associated with having a skilled birth attendant (OR 0.638, *p* = 0.007).

### 3.6 Facility delivery

A total of 5,667 women (90.1%) had a child under five years of age and information available about facility delivery (Table 4). Among these women, 5,250 (92.6%) delivered at a health facility. Adjusted for variables in the IPV analyses, having experienced IPV trended toward negative association with facility delivery but did not reach statistical significance (OR 0.846, *p* = 0.196).

## 4 Discussion

IPV is a significant public health concern in global women's health, with wide-ranging ramifications, including on prenatal and peripartum maternal healthcare. IPV in Kenya has been associated with young marital age, low wealth index, urban residence, depression, minimal educational attainment, drug and alcohol abuse, and higher risk of contracting HIV infection (28, 33, 34). Factors identified by our study that were associated with having experienced IPV in the past included age 25–49, emotional abuse, IPV exposure in girlhood, feeling safe in current relationship, mild or severe depression, an attitude supportive of wife beating, and partnership of any sort, including married monogamous, married polygamous, widowed, or separated. Previously identified risks not reflected in our results include the low wealth index; we assessed for an association between wealth quartile and risk of IPV and found no correlation. Past experience of IPV was associated with worse antenatal and perinatal health care, with expecting mothers being less likely to attend four or more prenatal care visits and have a skilled delivery attendant present at the birth.

Globally, the WHO estimates that 27% of women have had a lifetime experience of IPV (4). The 2022 Kenya DHS found that 34% of all Kenyan women aged 15 and older, and 51.1% of women living in Migori County, Kenya had experienced physical violence, while 13% of all Kenyan women aged 15–49, and 16.7% of women living in Migori County, Kenya had experienced sexual violence (31). A recent study of pregnant women in Kenya found that physical violence was the most common type of IPV experienced, at 78.6% of all IPV (56). Among women in Migori County, we found an IPV rate of 36.7%. A previous study of IPV from Lwala, published by Morris et al. (2022), found a lifetime prevalence of 60.3% in Migori county (28). In contrast, our study, which took place in 2021 and included additional geographic regions of Rongo sub-county, found that only 36.7% of the women of Migori county had experienced IPV. The difference in the DHS finding and our study may be attributed to differences in geographic coverage and ages sampled (44). Additionally, our survey generally provides more dense coverage and larger sample sizes compared to the DHS, which is designed to calculate rates for the entire country. As for the difference in IPV prevalence between the prior study from Morris et al. (2022) and our study, sample size and the increased geographic area serve as a possible explanation: the 2018 data used by Morris et al. included 873 women, while our 2021 administration of the survey reached 6,290 female respondents. Still, the decreased incidence of IPV from the 2018 survey is surprising given that the COVID-19 pandemic occurred in the interim; the lockdowns were associated with increases in the global IPV incidence, an increase which was corroborated by a 2020–2021 study of IPV in Kenya (57–59), but not a trend seen in our own data. This may be due, in part, to the rural setting of this study where community movement and interaction was not as limited. Additional research, including qualitative studies, are needed to further evaluate these differences.

Age group was found to be a significant factor in predicting the likelihood of past IPV experience, with women between 25 and 49 years being more likely to experience IPV than those aged 18–24 and those older than 50. This is consistent with the Kenyan DHS survey, which revealed that the highest percentage of women experiencing physical or sexual violence in the past 12 months are those between the ages of 25–49 (31). This finding highlights the vulnerability of women during their reproductive and child rearing years and emphasizes the importance of targeted interventions to address IPV in this age group.

Prior and current married/partnered status significantly increased the prevalence of IPV in our study, with women in monogamous or polygamous marriages, as well as those who were widowed, divorced, or separated, having higher odds of IPV experience. The most common perpetrator of physical violence found by the 2022 Kenya DHS was a current husband/intimate partner (54%) followed by a former husband/intimate partner (34%). The most common perpetrator of sexual violence was a current husband/intimate partner (71%) followed by a former husband/intimate partner (19%) (31). While our data does not stratify IPV experienced by the type of perpetrator, the Kenya DHS findings corroborate our finding that the past and present partnered women in our survey population were at higher risk. This underscores the need to address marital dynamics and intimate partnerships, power imbalances, and social norms that perpetuate violence within relationships. In fact, 12.1% of women surveyed in our study felt that wife-beating was “sometimes okay,” and women who held this belief were twice as likely to have experienced IPV. The actual number may be even higher, as the 2022 Kenya DHS reported that 46.2% of Kenyan women feel that wife-beating is sometimes justifiable, and 67.2% of women in Migori county feel wife beating is sometimes justifiable (31). This emphasizes the need for comprehensive efforts to document, understand, challenge, and change these harmful social and gender norms to promote gender equality and respect within communities. Community-wide accepting attitudes toward violence against women have been associated with increased rates of IPV perpetrated toward women, and these attitudes influence the community response toward instances of violence (60, 61). This provides potential avenues for interventions by government and civil-society-led organizations, such as engaging in norms change and proposing institutional changes. Campaigns that engage in norms change around IPV, targeted at both perpetrators and the general public, are a common method of mitigating IPV that has been used around the world, including in Kenya (62–64). Such campaigns include measures like collaborating with local government to display public anti-IPV infomercials, offering voluntary referrals to counseling programs for perpetrators, and implementing help lines for both victims and perpetrators; data collected during these campaigns shows them to be largely successful (63, 65). Still, other scholars have found that there is little empiric data to suggest meaningful reduction of IPV thus far, but that intentional, community-informed interventions to increase public awareness could certainly be beneficial (66).

Emotional abuse is a very common form of IPV—emotional abuse accounts for up to 67.8% of IPV experienced by pregnant women (56). Our study revealed a relationship between emotional abuse and IPV experiences, with women who were exposed to emotional abuse having ten times higher odds of experiencing IPV. This trend in emotional and physical abuse has been found to coexist in numerous prior studies (67, 68). Importantly, emotional abuse has been shown to be a predictor of future physical abuse (69, 70). The negative impact of emotional abuse independently, and its contributions to physical violence, suggest the importance of screening for emotional abuse among at-risk women in the interest of reducing IPV. Another potential benefit of screening for emotional abuse, or any sort of IPV, is prevention of IPV in future generations. Like in Morris et al. (2022), girlhood exposure to IPV was found to be associated with higher odds of IPV experience after age 18 in our study. This is also consistent with studies from other countries (61).

One unusual finding of our study is that women who felt safe in their current relationship were more likely to report a past or current history of IPV. Reasons for this finding may be multifactorial. First, the question “Do you feel safe at home?” has been found to only have a sensitivity of 8.8% when asked in a primary care setting, which suggests that there are women who respond “yes” to this question despite experiencing physical violence within their home or who do not view physical violence as a safety threat (71). Another potential explanation is that women who responded that they were currently feeling safe, but had increased odds of an IPV experience, were no longer in danger; perhaps they had left an abusive relationship.

The study also investigated the impact of lifetime experience of IPV on maternal healthcare, both prenatally and peripartum. Our analysis shows women who had experienced IPV were less likely to attend the recommended minimum of four antenatal care (ANC) visits during their most recent pregnancy. This finding is supported by literature investigating prenatal care for IPV-positive women (72). ANC visits are essential for monitoring maternal and fetal health, detecting complications, and providing necessary support and information. The lower utilization of ANC services among women experiencing IPV suggests barriers to accessing healthcare and underscores the importance of integrating IPV screening and support in maternal care settings. Furthermore, we found that women who had experienced IPV were less likely to have a skilled birth attendant during delivery. Skilled birth attendants play a crucial role in ensuring safe deliveries and reducing maternal and neonatal mortality, including mother to child transmission (MTCT) of HIV in HIV endemic areas like Migori County. The reduced likelihood of having a skilled birth attendant among women experiencing IPV indicates a gap in accessing necessary and sufficient maternal healthcare. Efforts should be made to enhance access to skilled birth attendants for this vulnerable population, ensuring the safest possible care during childbirth.

Further emphasizing the urgent importance of IPV interventions for improved maternal health outcomes are the health impacts on mother and child. Infants of IPV-positive mothers are at higher risk for pre-term birth, low birth weight, and neonatal death (73–75). Mothers with past experience of IPV are known to have increased prenatal and postpartum morbidity and mortality (15, 19, 76). This comes in the form of pregnancy-related morbidity such as vaginal bleeding, urinary tract infections, and pre-term labor as well as pregnancy-associated homicide and suicide. A concerning additional aspect of this issue is that co-occurring depression among expectant IPV-positive mothers has been shown to further worsen maternal and infant health outcomes (77–79). In our study, where 42.5% of women had a non-zero depression score, depression scores of mild and severe were both associated with increased odds of IPV experience. Given the intersectional nature of mental health, IPV, and pregnancy, programming surrounding these topics could positively impact the health of mother and child.

### 4.1 Limitations

The use of self-reported data and the cross-sectional design restrict the ability to establish causal relationships and may be subject to recall and social desirability biases. The study focused on a specific sub-county in Kenya, limiting the generalizability of the findings to other regions and countries. Furthermore, our data only includes findings regarding interpersonal violence perpetrated against women, which excludes the fact that IPV can be carried out by or against any gender. In addition, our data does not subdivide the type or gender of the perpetrator, which limits our ability to investigate trends in who is responsible for causing instances of IPV. To understand the underpinnings of IPV in Migori county, it is important to explore not only female attitudes toward wife-beating (already addressed by our survey), but also male attitudes. To address this limitation, future iterations of the survey will include both male and female respondents. The updated version of the survey is currently being administered by the Lwala Community Alliance. Finally, our data does not address the rates of IPV experienced by women during their pregnancy; we only examined lifetime IPV experience and its effect on pregnancy and maternity care. IPV experienced during a pregnancy could impact maternal and child outcomes and is an area that needs to be further explored. Despite the aforementioned limitations, this hyperlocal data is of relevance to our community-based NGO and findings gleaned from our analysis can contribute to other organizations' efforts of reducing the prevalence of IPV in their communities.

## 5 Conclusion

This study provides valuable insights into the prevalence of IPV and its impact on maternal healthcare in Migori County, Kenya. IPV exposure is associated with lower use of maternal care services and may lead to worse maternal health outcomes. This underscores the urgent need for comprehensive interventions that address social and gender norms perpetuating violence against women. The study findings can guide policy interventions and inform community responses to IPV, aiming to create safer and healthier environments for women in Migori County and beyond.

## Data Availability

The original contributions presented in the study are included in the article/Supplementary Material, further inquiries can be directed to the corresponding author.

## Appendix

**Table A1:** multivariate regression of factors associated with IPV, including crude and adjusted odds ratio.

Variable	COR (95% CI)	*P*-value	AOR (95% CI)	*P*-value
Region				
North Kamagambo	Ref		Ref	
East Kamagambo	1.163 (0.950, 1.424)	0.143	1.246 (0.971, 1.598)	0.084
Central Kamagambo	1 (0.816, 1.226)	0.999	0.855 (0.654, 1.119)	0.254
South Kamagambo	0.977 (0.794, 1.201)	0.823	0.956 (0.739, 1.237)	0.732
Central Kanyamkago	0.983 (0.798, 1.211)	0.871	0.928 (0.713, 1.207)	0.576
West Kanyamkago	**1.309** (**1.068, 1.603)**	**0**.**009****	1.093 (0.848, 1.41)	0.49
North Sakwa	1.048 (0.856, 1.284)	0.649	1.051 (0.817, 1.351)	0.701
Central Sakwa	0.959 (0.777, 1.183)	0.697	1.000 (0.77, 1.3)	0.998
Age				
18–24	Ref		Ref	
25–49	**1.464** (**1.306, 1.642)**	**<0**.**001*****	**1.208** (**1.045, 1.397)**	**0**.**011***
50+	**1.456** (**1.306, 1.642)**	**0**.**005****	1.137 (0.766, 1.688)	0.524
Religion				
SDA	Ref		Ref	
Catholic	1.16 (0.996, 1.351)	0.057	1.163 (0.96, 1.408)	0.124
Protestant	1.126 (0.979, 1.295)	0.097	1.044 (0.874, 1.247)	0.633
Roho	**1.302** (**1.124, 1.508)**	**<0**.**001*****	1.209 (0.998, 1.465)	0.053
vOther	1.115 (1.124, 1.508)	0.322	1.076 (0.821, 1.41)	0.595
Marital status				
Single	Ref		Ref	
Married monogamous/cohabitating	**2.695** (**1.124, 1.508)**	**<0**.**001*****	**2.152** (**1.426, 3.248)**	**<0**.**001*****
Married polygamous	**4.657** (**3.227, 6.719)**	**<0**.**001*****	**2.924** (**1.826, 4.683)**	**<0**.**001*****
Widowed/divorced/separated	**2.826** (**3.227, 6.719)**	**<0**.**001*****	**1.745** (**1.094, 2.786)**	**0**.**02***
Highest level of education				
No education	Ref		Ref	
Primary	0.793 (0.521, 1.208)	0.281	1.199 (0.631, 2.28)	0.579
Secondary+	**0.576** (**0.378, 0.879)**	**0**.**01****	0.957 (0.497, 1.843)	0.896
Experience of emotional abuse				
No	Ref		Ref	
Yes	**12.199** (**10.662, 13.958)**	**<0**.**001*****	**10.462** (**9.037, 12.112)**	**<0**.**001**
IPV exposure in girlhood				
No	Ref		Ref	
Yes	**3.792** (**3.390, 4.243)**	**<0**.**001*****	**2.525** (**2.202, 2.896)**	**<0**.**001**
Feels safe in current relationship				
Yes	Ref		Ref	
No	**0.827** (**0.726, 0.942)**	**0**.**004****	**0.722** (**0.609, 0.855)**	**<0**.**001**
Ever HIV tested				
No	Ref		Ref	
Yes	1.469 (0.836, 2.582)	0.181	1.124 (0.557, 2.269)	0.744
Depression Score				
None	Ref		Ref	
Mild	**2.057** (**1.813, 2.333)**	**<0**.**001*****	**1.482** (**1.269, 1.73)**	**<0**.**001*****
Moderate	**1.582** (**1.327, 1.885)**	**<0**.**001*****	1.015 (0.812, 1.269)	0.896
Moderately severe	**1.828** (**1.476, 2.264)**	**<0**.**001*****	0.995 (0.759, 1.305)	0.972
Severe	**4.128** (**2.784, 6.121)**	**<0**.**001*****	**2.403** (**1.429, 4.039)**	**0**.**001*****
Childhood mortality (last 5 years)				
No	Ref		Ref	
Yes	1.062 (0.681, 1.654)	0.792	1.29 (0.76, 2.189)	0.346
Attitude supportive of wife beating				
Never okay	Ref		Ref	
Sometimes okay	**2.314** (**1.986, 2.696)**	**<0**.**001*****	**2.002** (**1.651, 2.428)**	**<0**.**001*****
Currently pregnant				
No	Ref		Ref	
Yes	1.057 (0.891, 1.254)	0.522	1.134 (0.918, 1.401)	0.242
Wealth quartile				
Non-poor	Ref		Ref	
Vulnerable	**1.435** (**1.239, 1.663)**	**<0**.**001*****	0.998 (0.826, 1.206)	0.982
Poor	**1.34** (**1.155, 1.553)**	**<0**.**001*****	0.842 (0.689, 1.028)	0.091
Severely poor	**1.47** (**1.269, 1.704)**	**<0**.**001*****	0.846 (0.682, 1.048)	0.125

Bold values indicate statistically significant findings.

* denotes *p* ≤ 0.05.

** denotes *p* ≤ 0.01.

*** denotes *p* ≤ 0.001.

Appendix I describes the same information as Table 3, but includes the unadjusted odds ratio and its -value for reference.
